# Health-Related Effects of Home Nebulization With Glycopyrronium on Difficult-to-Treat Asthma: Post-Hoc Analyses of an Observational Study

**DOI:** 10.2196/17863

**Published:** 2020-04-29

**Authors:** Deepak Talwar, Salil Bendre

**Affiliations:** 1 Department of Pulmonology and Sleep Medicine Metro Centre for Respiratory Diseases Noida India; 2 Department of Chest Medicine KLS Memorial Hospital Mumbai India

**Keywords:** difficult-to-treat asthma, exacerbation, glycopyrronium, home nebulization, uncontrolled asthma, vibrating mesh nebulizer

## Abstract

**Background:**

Bronchial asthma remains a clinical enigma with poorly controlled symptoms or exacerbations despite regular use of inhaled corticosteroids. Home nebulization offers a simplified solution for the delivery of rescue and maintenance bronchodilators, which is especially true for patients with frequent exacerbations during management of uncontrolled or difficult-to-treat asthma.

**Objective:**

We aimed to assess the clinical impact and outcomes associated with home nebulization—delivered long-acting bronchodilators for uncontrolled or difficult-to-treat asthma.

**Methods:**

This observational, concurrent study was conducted with 60 patients at 2 centers during November 2018. Statistical analyses for prebronchodilator forced expiratory volume in one second (FEV1) and Global Initiative for Asthma (GINA) asthma control score in patients on long-acting bronchodilators and corticosteroids were conducted, with two-tailed *P* values <.05 considered statistically significant.

**Results:**

Per protocol analyses (53/60) for consecutive cases receiving home nebulization with long-acting bronchodilators and corticosteroids were conducted. The baseline demographics included a male-to-female ratio of 30:23 and mean values of the following: age, 60.3 years (SD 11.8 years); weight, 64 kg (SD 16.8 kg); FEV1, 43% (SD 16%); GINA asthma control score, 3.0 points (SD 0.8 points); serum eosinophil level, 4% (SD 3%); fractional exhaled nitric oxide (FeNO), 12.1 ppb (SD 6 ppb). Of the patients, 100% (53/53) had uncontrolled symptoms, 69.8% (37/53) had prior exacerbations, 100% (53/53) used formoterol/budesonide, and 75.5% (40/53) used glycopyrronium. The per protocol group (n=53) had significantly improved mean prebronchodilator FEV1 (23.7%, SD 29.8%; 0.46 L, SD 0.58 L; *P*<.001) and GINA asthma control score (2.1 points, SD 0.8 points, *P*<.001). At baseline, patients (n=40) receiving glycopyrronium/formoterol/budesonide (25/20/500 mcg) nebulization admixture had the following mean values: prebronchodilator FEV1, 38% (SD 15%); GINA asthma control score, 3.0 points (SD 0.8 points); reversibility, 12% (SD 6%); peripheral eosinophil level, 4% (SD 3%); FeNO, 12 ppb (SD 5.7 ppb). In the post hoc analyses, these patients had significantly improved mean prebronchodilator FEV1 of 27.7% (SD 26.2%; 0.54 L, SD 0.51 L; *P*<.001) at 8 weeks compared with baseline. At baseline, patients (n=13) receiving formoterol/budesonide (20/500 mcg) nebulization had the following mean values: FEV1, 55% (SD 12%); GINA asthma control score, 3.0 points (SD 1.2 points); reversibility, 14% (SD 7%); serum eosinophil level, 4% (SD 3%); FeNO, 13.3 ppb (SD 6.8 ppb). In the post hoc analyses, these patients showed a significant improvement in prebronchodilator FEV1 of 11.2% (SD 13.1%; 0.22 L, SD 0.25 L; *P*<.001) from baseline. Breathlessness of mild to moderate intensity was reported by 10 cases (10/53, 18.9%), with no other treatment-emergent adverse events or serious adverse events.

**Conclusions:**

Home nebulization remains a viable option for symptomatic difficult-to-treat asthma cases with frequent use of rescue medications. Glycopyrronium as add-on therapy offers a synergistic response in patients on corticosteroids with difficult-to-treat asthma.

**Trial Registration:**

Clinical Trial Registry of India CTRI/2018/11/016319; https://tinyurl.com/y78cctm3

## Introduction

Bronchial asthma remains a clinical enigma with high rates of morbidity and mortality. The Global Burden of Disease Study [1] highlighted the increasing trends in bronchial asthma, with 37.9 million people currently affected and an increase in the prevalence rate from 3.3% to 4.2%. Notwithstanding the current advances in the understanding of the disease topography or landscape involving the clinical phenotypes and related endotypes of Th2 and non-Th2 inflammation, most patients in real-world settings continue to have uncontrolled or difficult-to-treat asthma. According to a Dutch survey [2] among patients with bronchial asthma, 17% of cases had difficult-to-treat asthma despite a background of Global Initiative for Asthma (GINA) Step 4 or Step 5 therapy involving medium to high doses of combination inhaled corticosteroid and long-acting beta agonist (ICS/LABA). Poor symptom control in such cases is often related to the modifiable risk factors of incorrect inhaler technique, poor adherence, smoking, incorrect diagnoses, small airway disease (SAD), or non-Th2 inflammation that may require a complementary approach with nebulization or therapies involving long-acting muscarinic antagonists (LAMAs) [3].

In patients with severe asthma experiencing more than 2 exacerbations per year or hospitalization, the complementary role of LAMAs has often been considered. Similarly, LAMAs have complemented medium to high doses of ICS/LABA resulting in improved asthma control scores, especially for patients with severe exacerbation [4,5].

However, for most severe cases with uncontrolled asthma, the need for a simplified device to deliver rescue and maintenance bronchodilators administered at home or in ambulatory settings to treat or prevent moderate or severe exacerbations that requires a minimal inspiratory flow rate remains unmet. GINA recommends review of inhaler techniques at every step of asthma control, with due patient recognition and choice of inhaler devices including nebulizers for delivery of acute or maintenance medications [6,7].

A post hoc analysis by Morjaria et al [8] highlights the use of ICS/LABA as single maintenance and reliever therapy compared with PRN salbutamol for a highly significant attenuation in the rate of severe exacerbations, especially in patients with moderate to severe asthma, which is similar to the findings with tiotropium as observed by Kerstjens et al [9]. The clinical dilemma on the choice of therapies involving ICS or LAMAs is further dissected by the representation of bronchial asthma as a heterogenous condition involving eosinophilic or noneosinophilic asthma phenotypes. The noneosinophilic phenotype occurs in 50% of the severe asthma cases that are typified by biomarker assessments of peripheral eosinophil levels (300 eosinophils/μL) and fractional exhaled nitric oxide (FeNO; <30 ppb), wherein the role of LAMAs is usually suggested since these cases are nonresponsive to ICS, have a long standing history of uncontrolled or elderly asthma with airway remodeling, and likely have fixed airway obstruction changes suggestive of asthma-chronic obstructive pulmonary disease overlap or SAD. Usmani et al [10] observed an overall incidence of SAD of 50-60% among asthmatics, stating that its presence should not be overlooked or underestimated especially when managing severe asthma in real-world outpatient settings

Tiotropium has been clinically evaluated to offer ancillary control in noneosinophilic or paucigranulocytic asthma cases; this control may be correlated with its mechanistic action on the muscarinic receptors and related anti-inflammatory action. Glycopyrronium, an ultra-LAMA, offers quick, persistent, long-lasting bronchodilation and broad anti-inflammatory effects due to its stronger selectivity for M3 receptors as compared to other short-acting or long-acting LAMAs [11]. The anti-inflammatory action entails interleukin-1β and tumor necrosis factor-alpha cytokines for Th2-mediated and Th1-mediated inflammation control, as studied by Shen et al [12] and Kerwin et al [13], that may have relevance in the management of noneosinophilic or mixed granulocyte inflammatory phenotypes of severe asthma.

To further understand the clinical impact and role of home nebulization involving anticholinergics during the acute or maintenance phase of difficult-to-treat or uncontrolled asthma, we planned an observational, concurrent, multicentric study analyses.

## Methods

This observational, concurrent analysis (ie, the HRAA study) of home nebulization therapy was performed using 8 weeks of follow-up data from patients with uncontrolled asthma. The study was initiated following the review and approval of study documents by an independent institutional ethics committee at 2 centers across India. Consecutive cases of bronchial asthma receiving home nebulization in the last 3 weeks of November 2018 were enrolled using a 1:2 ratio of uncontrolled to difficult-to-treat cases, respectively, and followed for 8 weeks. For cases that had ongoing investigation, concurrent analyses were conducted for the missing details on the primary endpoint variables at 8 weeks. Patients receiving background therapy of inhaled ICS/LABA using a dry powder inhaler (DPI) or pressurized meter dose inhaler (pMDI) were directly switched to nebulization therapy with new-generation devices during study enrollment. The study was conducted as per the principles of the International Conference of Harmonization for Good Clinical Practice and Declaration of Helsinki while ensuring confidentiality of patient identifiers and written informed consent for the patients receiving support for the nebulizer devices.

The inclusion criteria included adult patients undergoing home nebulization for moderate to severe bronchial asthma that was uncontrolled despite receiving low or medium dose ICS/LABA as maintenance therapy and requiring an emergency department visit or frequent use of rescue medications. The exclusion criteria included currently a smoker; exposure to nonsmoking risk factors including cigarette smoke and biomass or occupational hazards; and the need for long-term oral corticosteroids, leukotriene receptor antagonists, or antihistamine combinations. Cases of chronic obstructive pulmonary disease and asthma-chronic obstructive pulmonary disease overlap were excluded based on spirometry assessment for obstructive airway disease with demonstration of reversibility involving a change in forced expiratory volume in one second (FEV1) >12% and >200 mL following salbutamol inhalation

Per protocol analyses were conducted with patient records with ≥1 follow-up visit for primary endpoints involving improvements in post-bronchodilator FEV1 and GINA asthma control score at 4 and 8 weeks.

Primary analyses for clinical cases were performed to assess asthma control status with symptomatic assessment using the GINA symptom scale for daytime and nighttime symptoms and activity limitation. As per the GINA asthma control symptom assessment, asthma control was defined as well-controlled, partly controlled, or uncontrolled, with total scores of 0, 1-2, and 3-4, respectively, at baseline, 4 weeks, and 8 weeks (follow-up). Difficult-to-treat cases were defined as uncontrolled asthma for patients receiving GINA recommended Step 4 or Step 5 regimens involving inhaled ICS/LABA combination that may have been optimized for treatment adherence or compliance and comorbidities as per the prescription records.

The primary study endpoints included the mean change at 8 weeks in prebronchodilator FEV1, as assessed using spirometry, and GINA asthma control score especially for daytime symptoms, nighttime symptoms, and activity limitations. A secondary endpoint was treatment-emergent adverse events at 8 weeks. The safety observations included treatment-emergent adverse events and were risk stratified as mild, moderate, or severe for any treatment modification, withdrawal, or referral for hospitalization. The National Coordination Centre, PvPI (India) was notified of all serious adverse events observed during the concurrent analyses.

This observational study was conducted to explore the current utilization and impact of home nebulization on the management of difficult-to-treat asthma; we determined an adequate sample size for the primary analyses involving Student’s *t* tests for continuous and categorical variables. We planned and performed descriptive analyses for patient demographic variables and Student *t* tests to compare the primary efficacy variables including prebronchodilator FEV1 and GINA asthma control scores between home nebulization with ICS/LABA and home nebulization with ICS/LABA with anticholinergics. Primary and post hoc statistical analyses involving categorical and numerical data were carried out using Fisher exact tests and Student *t* tests in QuickCalcs GraphPad Prism (version 7.05; San Diego, CA). Two-tailed *P* values <.05 were considered statistically significant. Descriptive statistics were used to assess treatment-emergent adverse events at 8 weeks.

## Results

**Figure 1:**
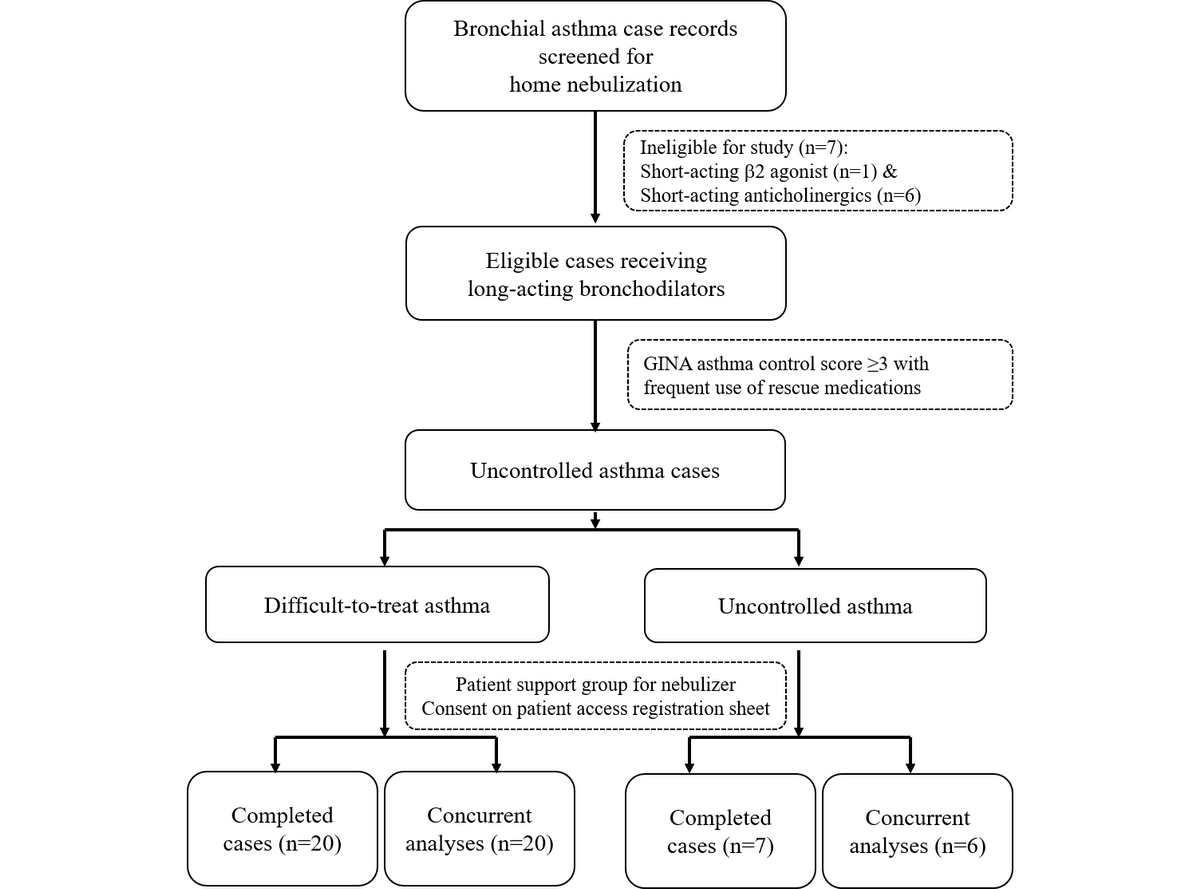
Patient analyses flow chart for this drug-utilization, case-control, observational clinical study.

In this observational, concurrent study, 60 consecutive cases undergoing home nebulization with 8 weeks of follow-up records were analyzed. In the control arm, 7 patients were excluded from the per protocol analyses due to maintenance therapy involving inhaled corticosteroids with levosalbutamol (n=1) or combination salbutamol/ipratropium (n=6; Figure 1).

Subsequent per protocol analyses were performed for 53 home nebulization prescriptions with long-acting bronchodilators including formoterol/budesonide with (n=40) or without (n=13) glycopyrronium. The choice of long-acting anticholinergic was assessed by the physician for the background use of short-acting anticholinergics in the acute phase and status as difficult-to-treat asthma after assessing compliance and adherence to ICS/LABA therapy. Baseline demographic characteristics included cases with severe uncontrolled asthma that were elderly with confirmed reversibility following observation of low FeNO levels following prior use of ICS/LABA with pMDI or DPI inhaler devices (Table 1).

All the patients with uncontrolled asthma were symptomatic (53/53, 100%) before referral to the emergency department or a hospital for persistent symptoms or exacerbation. Patients on a nebulized formoterol/budesonide (20/500 mcg) formulation administered twice daily were assessed as nonadherent to background therapy delivered by DPI or pMDI. On the other hand, patients assessed as difficult-to-treat cases after evaluation for concomitant comorbidities and compliance were prescribed add-on therapy of nebulized glycopyrrolate (25 mcg), which was co-administered with ICS/LABA as a 4-mL formulation for inhalation.

The mean GINA asthma control scores were assessed from the records for daytime symptoms, nighttime symptoms, activity limitation, and rescue medication use at baseline and 8 weeks for all cases (Textbox 1). The responses for each component are scored as 1 (Yes) or 0 (No). Total scores of 3-4, 1-2, and 0 at each visit indicate uncontrolled, partly controlled, and well-controlled asthma, respectively. For the patients receiving long-acting anticholinergic add-on therapy, glycopyrrolate (25 mcg) was delivered with formoterol/budesonide (20/500 mcg) as a 4-mL admixture and administered twice a day with a vibrating mesh or compressor air nebulizer over a period of 10 minutes.

**Table 1:** Baseline demographics for the per protocol analysis group (n=53).

Parameters			Results, n (%)
**Gender**			
	Male	30 (56.6)	
	Female	23 (43.4)	
Age (years)		60.4 (11.8)^a^	
Weight (kg)		64 (16.8)^a^	
FEV1^b^ (%)		43 (16)^a^	
FEV1 (L)		1.01 (0.42)^a^	
Reversibility (%)		13 (6)^a^	
History of a hospitalization or emergency department visit in the last year		37 (69.8)	
FeNO^c^ (ppb)		12.1 (6)^a^	
Peripheral eosinophil (%)		4 (3)^a^	
GINA^d^ asthma control score		3.0 (0.8)^a^	
Uncontrolled asthma with frequent rescue medication use		53 (100)	
Prior pMDI^e^ use		16 (30.2)	
Prior DPI^f^ use		37 (69.8)	
Oral xanthine use		33 (62.3)	
**Comorbidities**			
	Hypertension	21 (39.6)	
	ASCVD^g^	3 (5.7)	
	Bronchiectasis	5 (9.4)	
	ARDS^h^	5 (9.4)	
	Atopy	5 (9.4)	
**Treatment**			
	Nebulized glycopyrronium/formoterol/budesonide (25/20/500 mcg)	40 (75.5)	
	Nebulized formoterol/budesonide (20/500 mcg)	13 (24.5)	

^a^Mean (SD).

^b^FEV1: forced expiratory volume in one second.

^c^FeNO: fractional exhaled nitric oxide.

^d^GINA: Global Initiative for Asthma.

^e^pMDI: pressurized meter dose inhaler.

^f^DPI: dry powder inhaler.

^g^ASCVD: atherosclerotic cardiovascular disease.

^h^ARDS: acute respiratory distress syndrome.

**Global Initiative for Asthma (GINA) asthma symptom scale score was assessed at each visit to determine well-controlled, partly controlled, or uncontrolled asthma. The responses for each component are scored as 1 (Yes) or 0 (No).**

- Daytime asthma symptoms >2 times/week
- Activity or exercise limited by asthma
- Waking during any night due to asthma
- Rescue medication (>2 times/week)

### Efficacy Variables

Per protocol analyses for the overall group (n=53) of prebronchodilator FEV1 and the total GINA asthma control scores for daytime and nighttime symptoms, activity limitation, and use of rescue medications at 8 weeks were significantly improved, by a mean 23.7% (SD 29.8%; 0.46 L, SD 0.58 L; *P*<.001) and 2.1 points (SD 0.8 points, *P*<.001), respectively.

The subgroup (n=40) receiving the glycopyrrolate/formoterol/budesonide (25/20/500 mcg) nebulizing solution admixture had a mean baseline FEV1 of 38% (SD 15%), mean reversibility of 12% (SD 6%), mean peripheral eosinophil level of 4% (SD 3%), and mean FeNO of 12 ppb (SD 5.7 ppb). In the post-hoc analyses, this subgroup had significant improvement in prebronchodilator FEV1 at 8 weeks, by a mean 27.7% (SD 26.2%; 0.54 L, SD 0.51 L; *P*<.001).

The subgroup (n=13) receiving home nebulization with formoterol/budesonide (20/500 mcg) nebulizing solution had a mean baseline FEV1 of 55% (SD 12%), mean reversibility of 14% (SD 7%), mean peripheral eosinophil level of 4% (SD 3%), and a mean FeNO of 13.3 ppb (SD 6.8 ppb). Similarly, this subgroup showed significant improvement in FEV1 at 8 weeks, by a mean 11.2% (SD 13.1%; 0.22 L, SD 0.25 L; *P*<.001).

At 8 weeks, the change in pre-bronchodilator FEV1 from baseline was significant in the group receiving nebulized formoterol/budesonide plus glycopyrronium add-on therapy (*P*<.001), compared with baseline (Table 2). Both subgroups withstood the test of interaction while demonstrating statistically significant responses or improvement in FEV1 and GINA asthma control score at the end of the 8-week observation period (*P*<.001), compared with baseline.

**Table 2:** Change in assessment values at 8 weeks, compared with baseline, for the overall group and by nebulized admixture.

Assessment	Nebulized ICS^a^ + bronchodilators^b^ (n=53)		Nebulized formoterol/budesonide + glycopyrronium (n=40)		Nebulized formoterol/budesonide (n=13)		
	Change	*P* value^c^	Change	*P* value^c^		Change	*P* value^c^
Pre-bronchodilator FEV1^d^ (L), mean (SD)	0.46 (0.58)	<.001	0.54 (0.51)	<.001		0.22 (0.25)	<.001
GINA^e^ asthma control score (points), mean (SD)	–1.8 (0.8)	<.001	–1.8 (1.0)	<.001		–1.8 (1.0)	<.001

^a^ICS: inhaled corticosteroid.

^b^beta 2 agonists or anticholinergics.

^c^Compared with baseline.

^d^FEV1: forced expiratory volume in one second.

^e^GINA: Global Initiative for Asthma.

Patient compliance with the admixture procedure was assessed and confirmed (100%) at every visit by the investigator based on verbal affirmation from the patient before administration in the home setting.

### Safety Analyses

During the 8-week observation period, 10 cases had a single episode of breathlessness (10/53, 18.9%) that required rescue medication consisting of short-acting beta agonists (3/53, 5.7%) or short-acting muscarinic antagonists (7/53, 13.2%). These cases of breathlessness were noted in cases of uncontrolled asthma receiving home nebulization (3/20, 15%) and/or concomitant xanthines (7/20, 21%), with no significant difference in the consumption of rescue medication (*P*=.72) between the groups.

No anticholinergic nor cardiovascular events or symptoms were noted with the use of long-acting bronchodilators during home nebulization during the observation period.

There were no other treatment-emergent adverse events or serious adverse events noted that required treatment modification or discontinuation of long-acting bronchodilator home nebulization therapy.

## Discussion

This real-world, observational study of home nebulization highlights the clinical impact and utilization of this strategy for cases of difficult-to-treat or uncontrolled asthma while delivering nebulized long-acting bronchodilators for symptomatic patients with severe airflow limitation.

Asthma is a heterogenous condition consisting of several phenotypes including eosinophilic and noneosinophilic or paucigranulocytic variants that usually respond to targeted therapy or symptomatic therapy with LAMAs. For patients with bronchial asthma and moderate to severe exacerbations, LAMAs offer complementary actions such as those highlighted by Kerstjens et al [9] for tiotropium and Virchow et al [14] for glycopyrronium. The current study conforms to the clinical approach described by those authors and describes the impact of LAMA add-on therapy for patients with bronchial asthma and moderate to severe exacerbations, with a clinically significant improvement in prebronchodilator FEV1 of 27.7% (SD 26.2%; 0.54 L, SD 0.51 L) at 8 weeks, when compared with baseline. However, these results assume significance since the all the cases were assessed for noneosinophilic or mixed granulocytic phenotype markers, including FeNO, before reversibility was confirmed.

Vibrating mesh nebulizers represent the new generation of inhaler devices that are compact, portable, noiseless, and convenient. They offer optimal lung deposition with tidal breathing while obviating the need for breath holding common with conventional devices with or without the use of spacers, thereby minimizing nonadherence and improving compliance in patients with physical or cognitive deficits. In this study, patient compliance and adherence were assessed as complete (100%), again highlighting the convenience of nebulization therapy in difficult-to-control cases where the adherence rates are usually inadequate, as reported by other epidemiological studies [3]. In this line, GINA further recommends customization or individualization of patient care at every step of asthma control, by taking into account self-assessed symptom control status, comorbidities, patient behavior or phenotypic characteristics, and preferences for a simplified unified inhalational device that can have an incremental impact on compliance and adherence to therapy, especially with the ultracompact mesh nebulizers [7].

These results are the first to highlight the likely clinical role of a home nebulization strategy to deliver long-acting maintenance bronchodilators including glycopyrronium for difficult-to-treat asthma or noneosinophilic asthma (NEA). It is estimated that around 50% of asthmatic patients are of the NEA phenotype, which can be neutrophilic or paucigranulocytic. Paucigranulocytic asthma cases usually have a lower incidence of atopy with airway hyperresponsiveness or reversibility as compared to eosinophilic asthma, again lending credibility to the clinical correlate with SAD with fixed airway obstruction due to remodeling effects [10,15-17]. In the post hoc analyses for the subgroup receiving the glycopyrronium nebulizing solution, none of the cases were atopic and had stable peripheral eosinophil levels (mean 4%, SD 3%) and FeNO (mean 12 ppb, SD 5.7 ppb), which indicated that they likely had the mixed granulocytic inflammatory or NEA phenotype that may not be responsive to anti-immunoglobulin E or other biologics that are directed towards management of severe eosinophilic asthma, as suggested by Holguin et al [17].

### Limitations

The study results are limited by the retrospective nature with concurrent analyses of severe asthma cases receiving home nebulization with long-acting bronchodilators involving beta 2 agonists and/or long-acting anticholinergics. Post hoc analysis on the greater clinical improvement in lung function with LAMA or glycopyrronium add-on therapy was likely to be confounded by the underlying patient demographic variables for NEA or SAD. This requires further validation through active controlled trials.

However, this study highlights the clinical feasibility and impact of the early initiation of home nebulization for clinically symptomatic cases of uncontrolled asthma that have been optimized for treatment adherence and compliance to conventional inhaler therapies but requiring frequent use of rescue medications. The study also explores the clinical role of LAMA or glycopyrronium add-on therapy for difficult-to-treat asthma cases with little or no evidence of Th2 inflammation or history of atopy. These results require further validation through active controlled trials for assessment of glycopyrronium add-on therapy in difficult-to-treat NEA or mixed granulocytic inflammatory phenotypes that remain unexplored to this date despite the current availability of study publications on LAMAs [9,14].

### Conclusion

Home nebulization with new-generation vibrating mesh nebulizers remains a clinically feasible option for patients with severe asthma and uncontrolled symptoms. It simplifies treatment administration and strategies for adherence to prevent or maintain remission rates in these cases, as highlighted by GINA. A glycopyrronium add-on strategy offers bronchodilation that is clinically meaningful, especially for patients with difficult-to-treat asthma with moderate to severe exacerbations.

## Abbreviations

DPI: dry powder inhaler
FeNO: fractional exhaled nitric oxide
FEV1: forced expiratory volume in one second
GINA: Global Initiative for Asthma
ICS/LABA: inhaled corticosteroid and long-acting beta agonist
LAMA: long-acting muscarinic antagonist
NEA: noneosinophilic asthma
pMDI: pressurized meter dose inhaler
SAD: small airway disease
